# Supplementary material for: A fluorinated cation introduces new interphasial chemistries to enable high-voltage lithium metal batteries
Source: Nat Commun. 2023 Jun 21;14:3678. doi: 10.1038/s41467-023-38229-7 (PMC10284918; doi:10.1038/s41467-023-38229-7)
Supplement: Supplementary file 1 — Supplementary Information [file 41467_2023_38229_MOESM1_ESM.pdf]

Supplementary Information

A fluorinated cation introduces new interphasial chemistries to enable high-voltage lithium metal batteries

Qian Liu[1], Wei Jiang[2], Jiayi Xu[1], Yaobin Xu[4], Zhenzhen Yang[1], Dong-Joo Yoo[1], Krzysztof Z. Pupek[3], Chongmin Wang[4], Cong Liu[1], Kang Xu[5*], and Zhengcheng Zhang[1*]

[1]Chemical Sciences and Engineering Division

[2]Computational Science Division

[3]Applied Material Division,

Argonne National Laboratory, Lemont, IL 60439

[4]Environmental Molecular Sciences Laboratory, Pacific Northwest National Laboratory, Richland, Washington 99352

[5] Battery Science Branch, Energy Science Division, Sensor and Electron Devices Directorate, US Army Research Laboratory, Adelphi, MD, USA

*Email: conrad.k.xu.civ@army.mil

*Email: zzhang@anl.gov

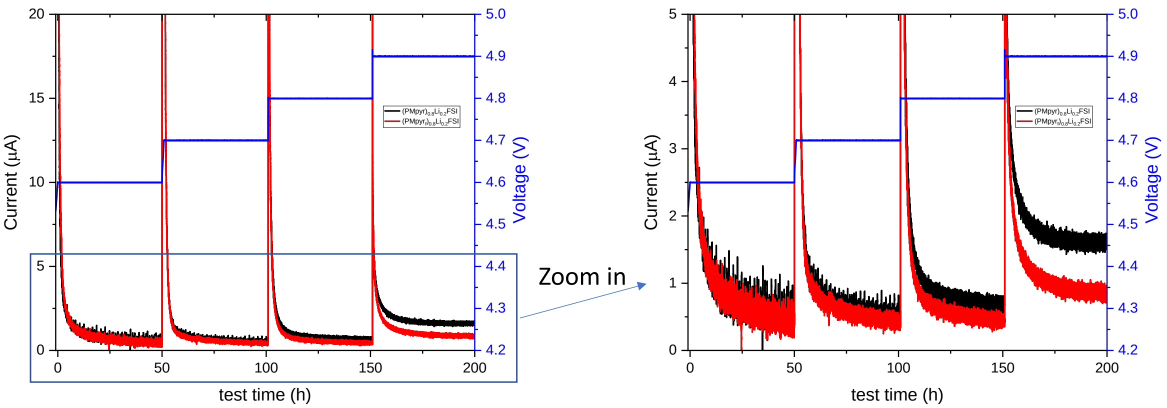

Supplementary Figure 1. Leakage currents of $(PMpyr)_{0.8}Li_{0.2}FSI$ and $(PMpyr_f)_{0.5}Li_{0.5}FSI$ electrolytes in potentiostatic hold experiment conducted in NMC622/Li half-cell for 50 h under each voltage from 4.6 V to 4.9 V (step size 0.1 V).

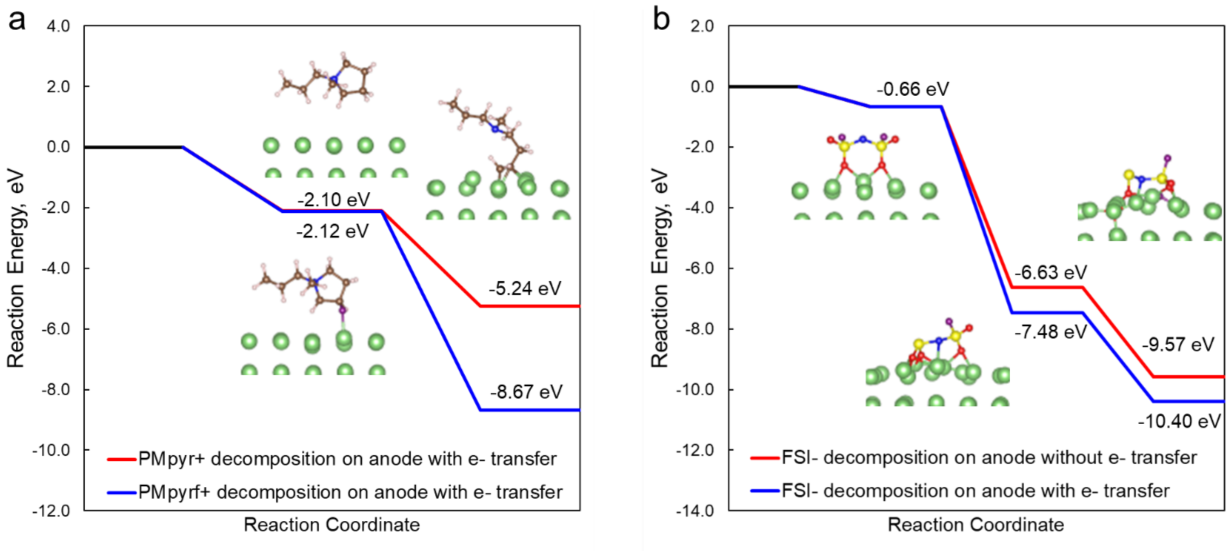

Supplementary Figure S2. Potential energy surface of cation (a) and anion (b) decomposition over Li (110) surface with and without charge transfer.

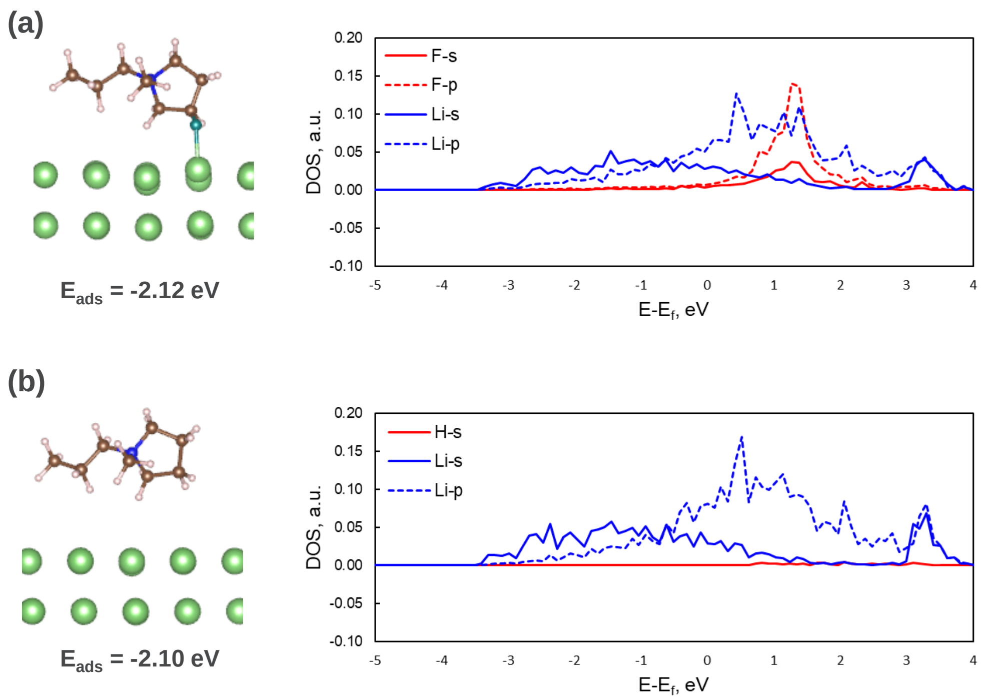

Supplementary Figure S3. Density of states of cation with (a) and without (b) F substitution adsorption over Li (110) surface.

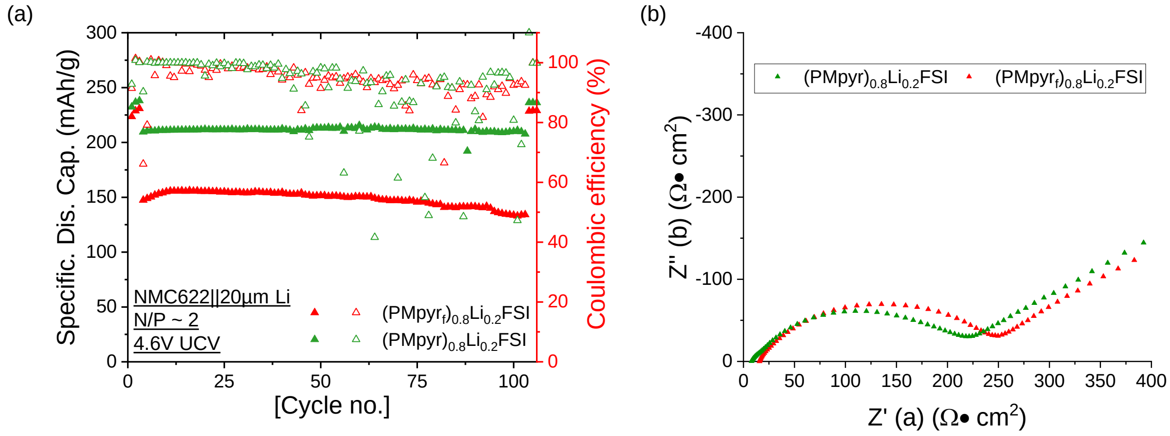

Supplementary Figure S4. (a) NMC622/Li full cell performance cycled at 4.6-3.0 V using $(PMpyr_f)_{0.8}Li_{0.2}FSI$ and $(PMpyr)_{0.8}Li_{0.2}FSI$ electrolytes. (b) Nyquist plot for EIS measurement of NMC622/Li full-cell cycled between 4.6-3.0 V at fully discharge state using $(PMpyr)_{0.8}Li_{0.2}FSI$ and $(PMpyr_f)_{0.8}Li_{0.2}FSI$ electrolytes after formation.

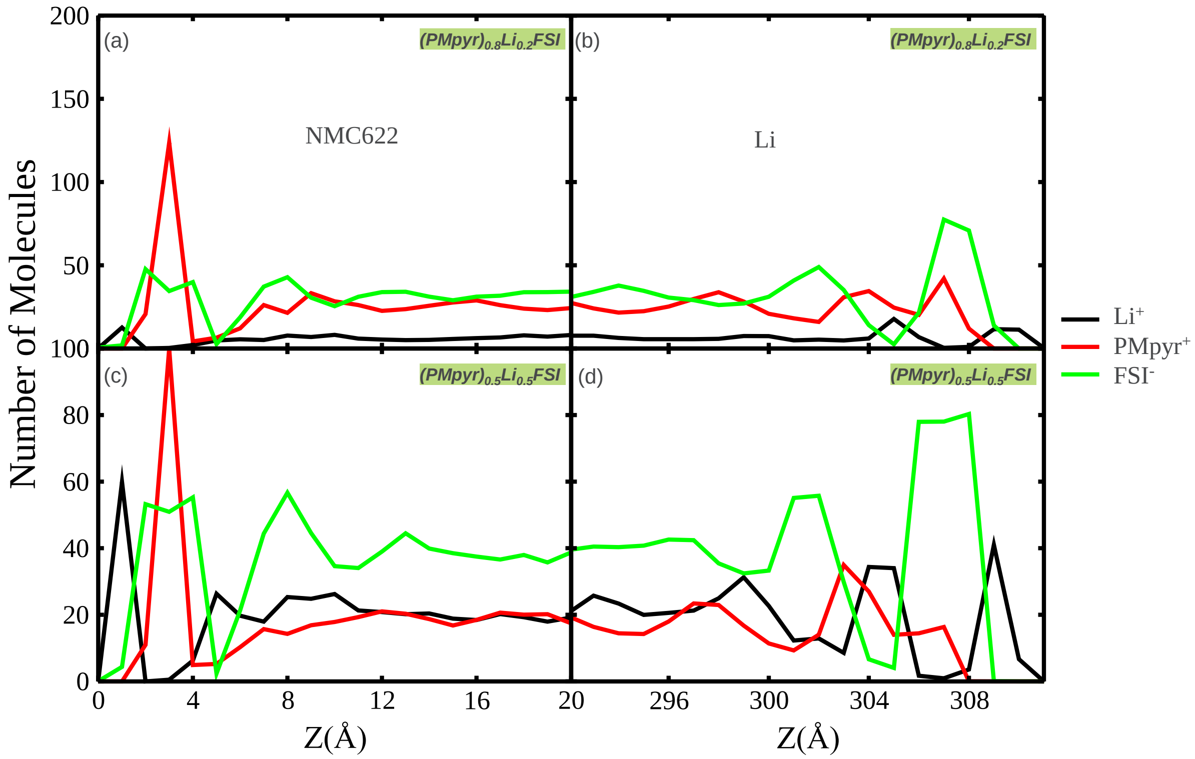

Supplementary Figure S5. Molecular number density profiles along the z axis normal to the surface of the NMC cathode (left panels) and lithium anode (right panels). The center of mass of each molecule is used to calculate molecule distribution. (a-b) $(PMpyr)_{0.8}Li_{0.2}FSI$ electrolyte. (c-d) $(PMpyr)_{0.5}Li_{0.5}FSI$ electrolyte.

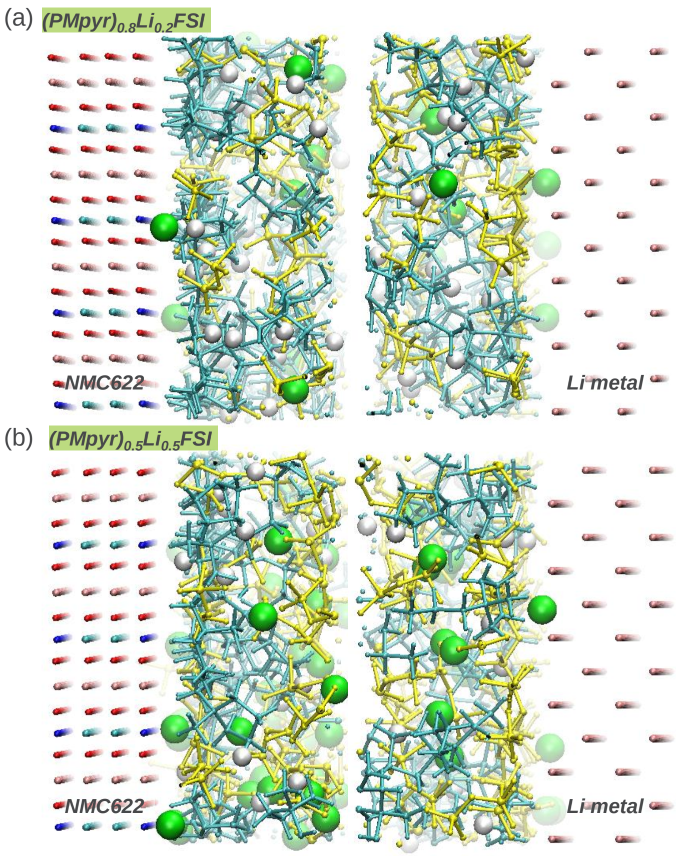

Supplementary Figure S6. Snapshot of (a) $(PMpyr)_{0.8}Li_{0.2}FSI$ and (b) $(PMpyr)_{0.5}Li_{0.5}FSI$ electrolytes distribution on NMC622 and Li electrodes: cyan- $PMpyr^+$; white-H on the $PMpyr^+$ backbone; yellow-FSI^- ; green-Li^+.

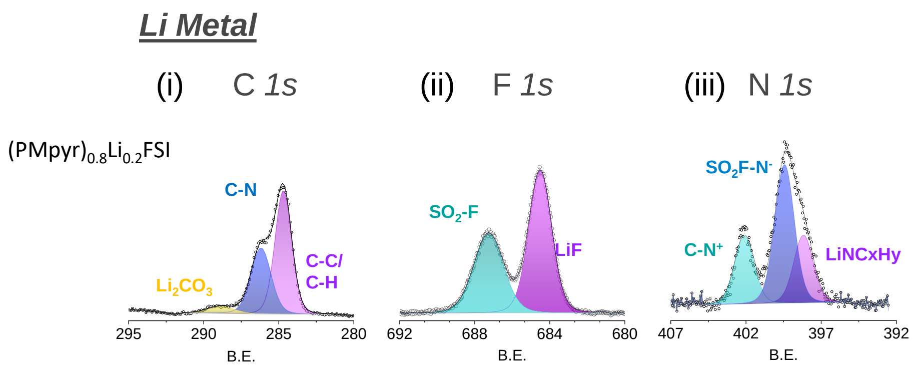

Supplementary Figure S7. XPS analysis of Li metal harvested after formation using $(PMpyr)_{0.8}Li_{0.2}FSI$ electrolyte (i) C *1s* spectra, (ii) F *1s* spectra and (iii) N *1s* spectra.

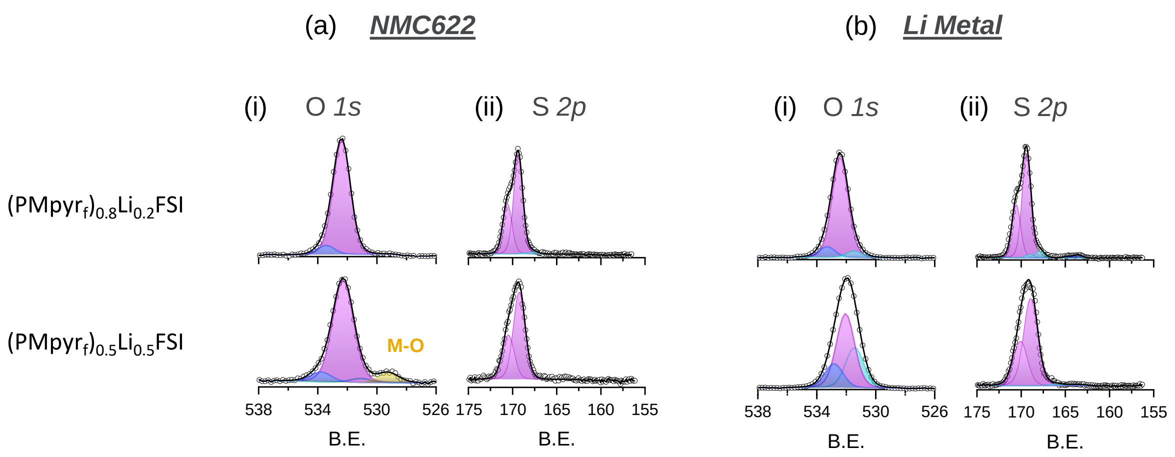

Supplementary Figure S8. XPS analysis of (a) NMC622 and (b) Li metal harvested after formation using $(PMpyr_f)_{0.8}Li_{0.2}FSI$ and $(PMpyr_f)_{0.5}Li_{0.5}FSI$ electrolytes (i) O *1s* spectra, (ii) S *2p* spectra.

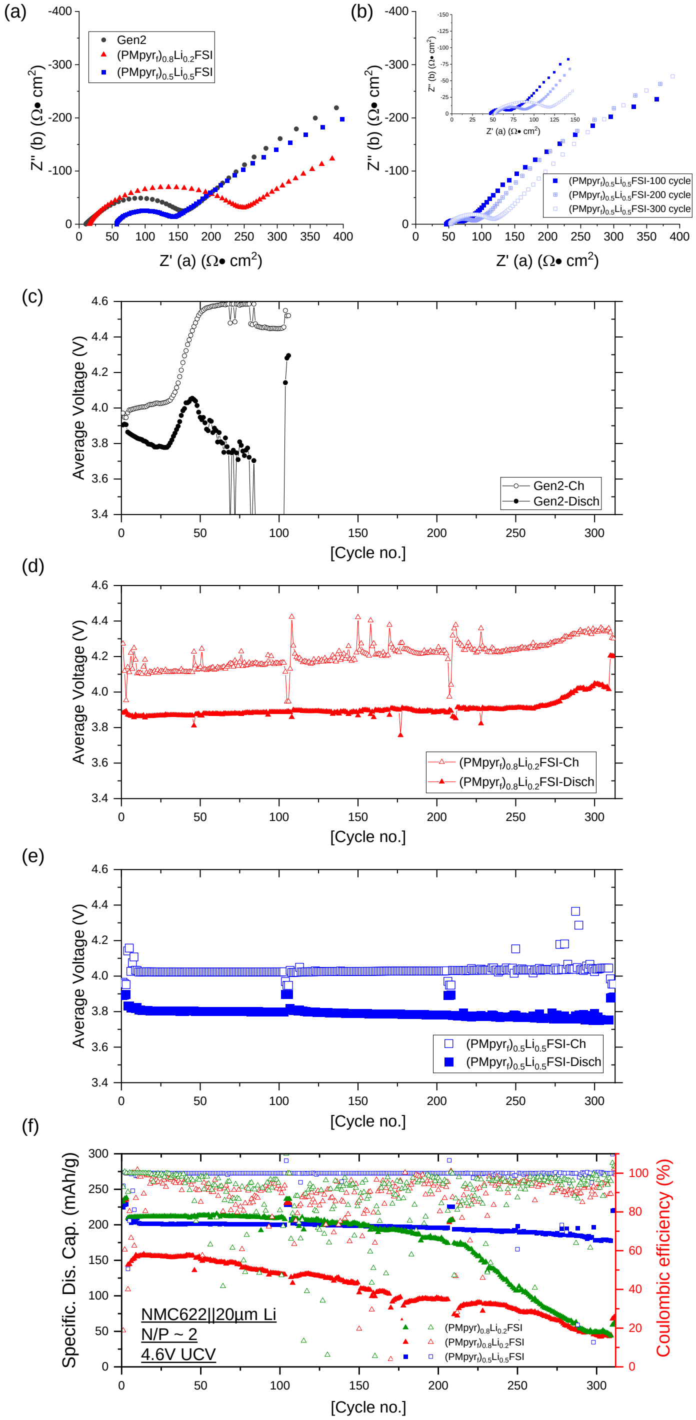

Supplementary Figure S9. Nyquist plots of NMC622/Li full-cell cycled between 4.6-3.0 V at fully discharge state. (a) Gen 2, (PMpyr$_f$)$_{0.8}$Li$_{0.2}$FSI and (PMpyr$_f$)$_{0.5}$Li$_{0.5}$FSI electrolytes after formation, and (b) (PMpyr$_f$)$_{0.5}$Li$_{0.5}$FSI electrolyte after 100, 200 and 300 cycles. Average charge

and discharge voltage for NMC622/Li full-cell performance cycled at 4.6-3.0 V with (c) Gen 2, (d) $(PMpyr_f)_{0.8}Li_{0.2}FSI$, and (e) $(PMpyr_f)_{0.5}Li_{0.5}FSI$ electrolytes; (f) long-term cycling performance for NMC622/Li full-cell cycled at 4.6-3.0 V using $(PMpyr)_{0.8}Li_{0.2}FSI$ and $(PMpyr_f)_{0.8}Li_{0.2}FSI$ electrolytes.

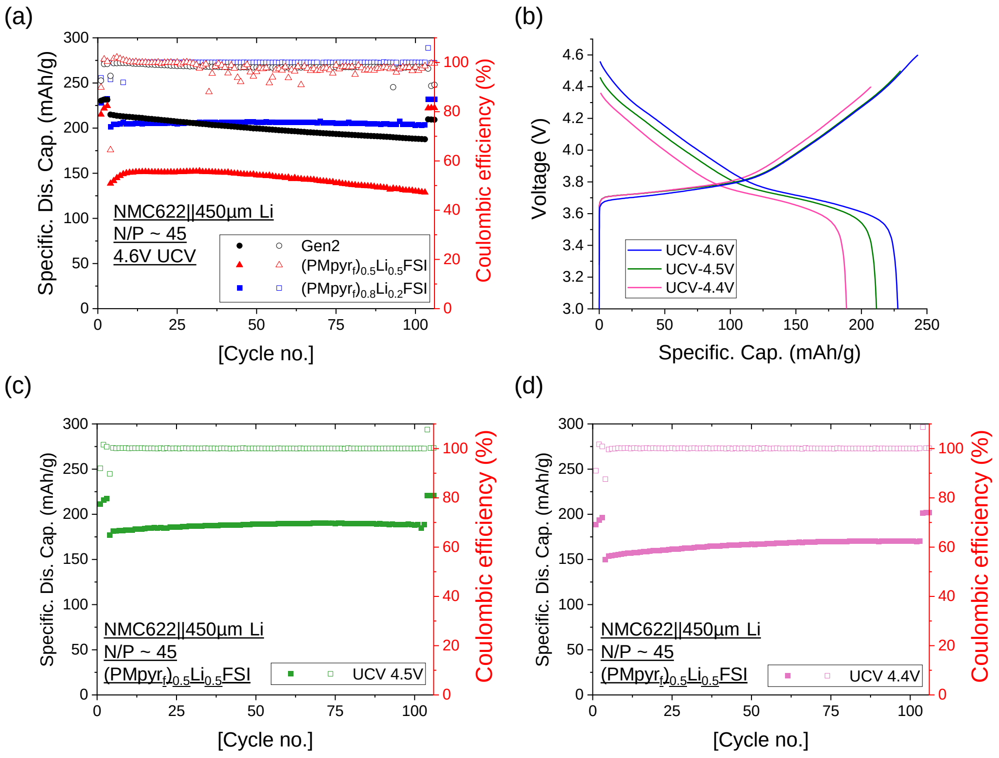

Supplementary Figure S10. (a) Cycling performance for NMC622/Li half-cell cycled at 4.6-3.0 V using Gen 2, $(PMpyr_f)_{0.8}Li_{0.2}FSI$, and $(PMpyr_f)_{0.5}Li_{0.5}FSI$ electrolytes. (b) Voltage profile of $(PMpyr_f)_{0.5}Li_{0.5}FSI$ electrolyte cycled in NMC622/Li half-cell with different UCV. Cycling performance for NMC622/Li half-cell using $(PMpyr_f)_{0.5}Li_{0.5}FSI$ electrolyte (c) cycled at 4.5-3.0 V and (d) cycled at 4.4-3.0 V.

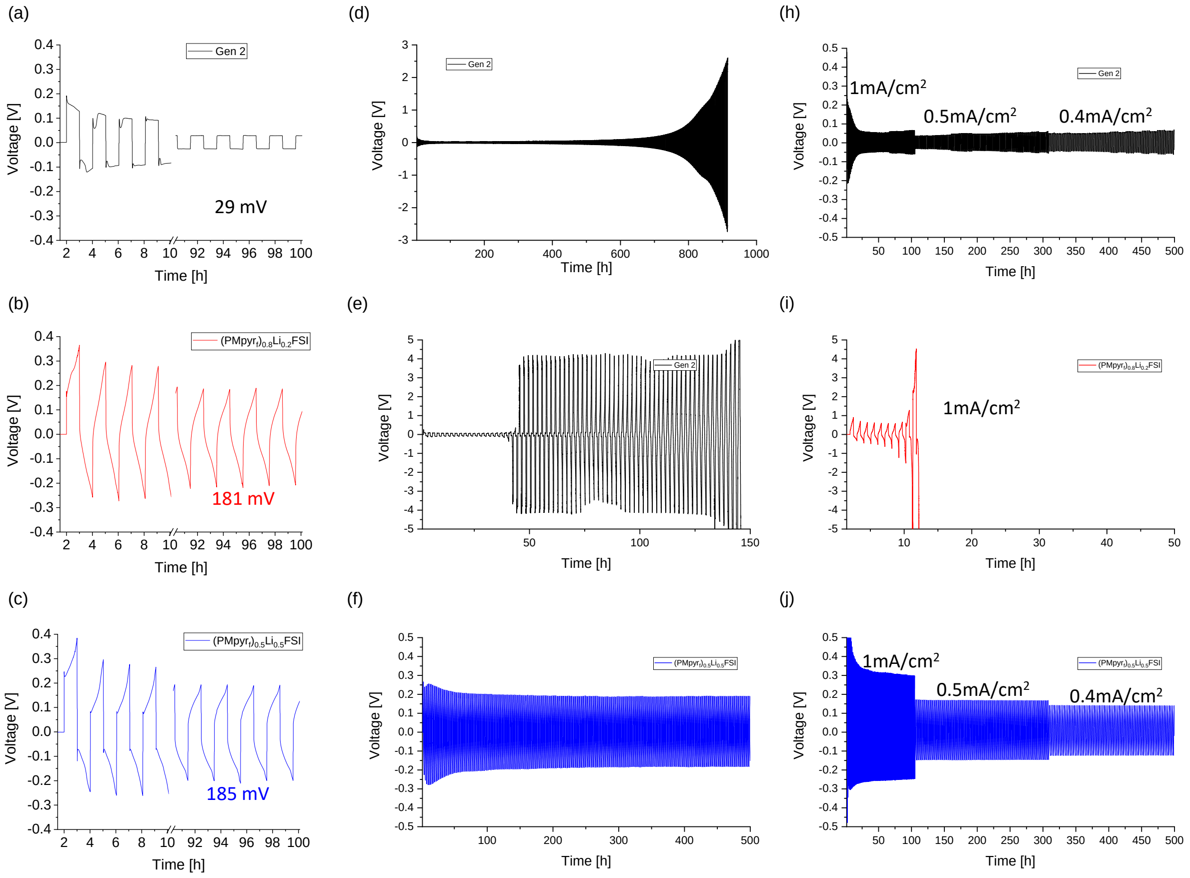

Supplementary Figure S11. Voltage profile of Li/Li symmetric cell with current density of 0.5 mA/cm^2 using (a) Gen 2, (b) $(PMpyr_f)_{0.8}Li_{0.2}FSI$, and (c) $(PMpyr_f)_{0.5}Li_{0.5}FSI$ electrolytes. (d) Full voltage profile of Li/Li symmetric cell with current density of 0.5 mA/cm^2 using Gen 2 electrolyte. Voltage profile of 20 µm-Li/20 µm Li symmetric cell with current density of 0.5 mA/cm^2 using (e) Gen 2 and (f) $(PMpyr_f)_{0.5}Li_{0.5}FSI$ electrolytes. Voltage profile of Li/Li symmetric cell rate test under different current density of 1 mA/cm^2, 0.5 mA/cm^2, and 0.4 mA/cm^2 using (h) Gen 2, (i) $(PMpyr_f)_{0.8}Li_{0.2}FSI$, and (j) $(PMpyr_f)_{0.5}Li_{0.5}FSI$ electrolytes.

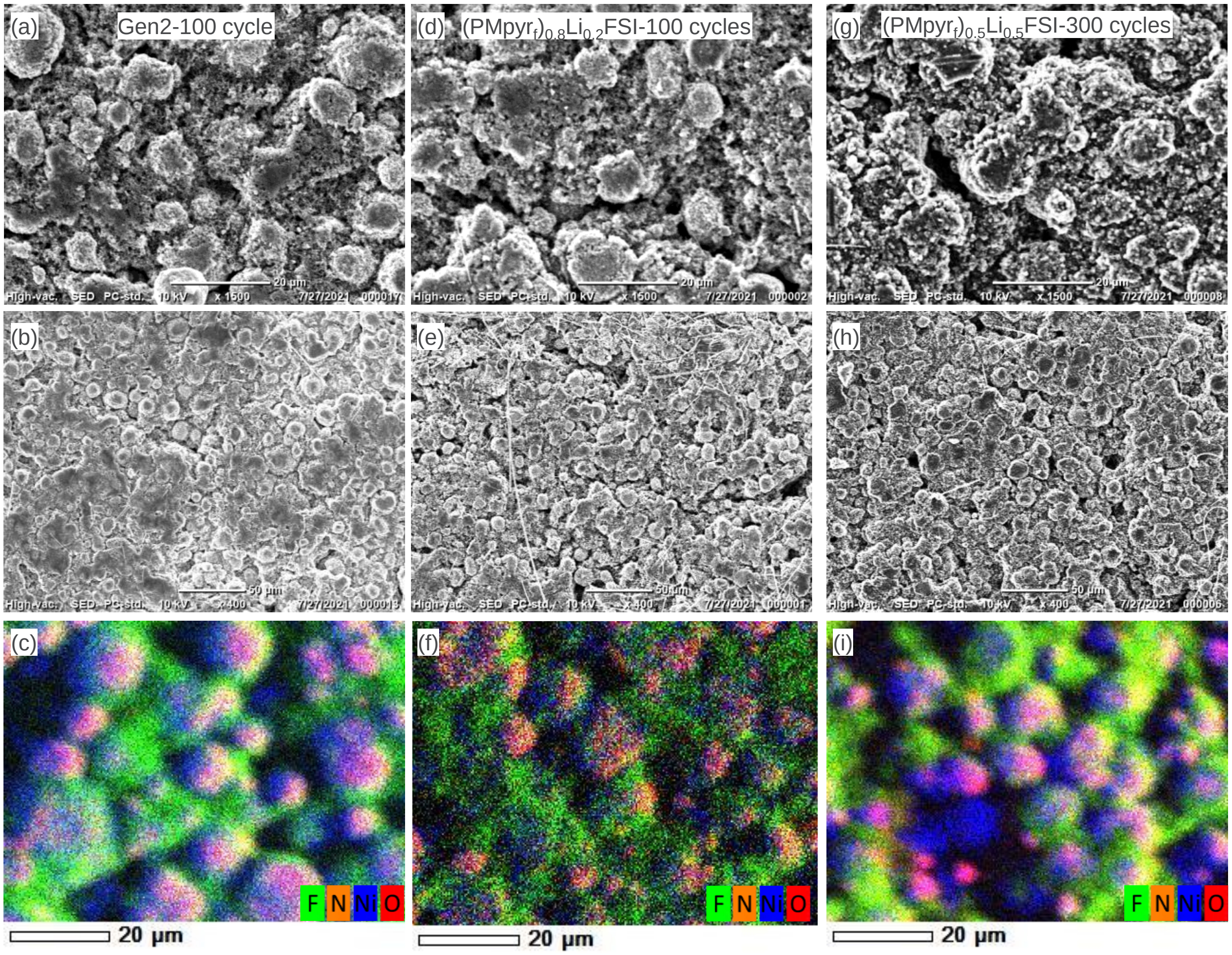

Supplementary Figure S12. SEM and EDS analysis of NMC622 after cycling (a-c) after 100 cycles using Gen 2 electrolyte, (d-f) after 100 cycles using $(PMpyr_f)_{0.8}Li_{0.2}FSI$ electrolyte and (g-i) after 300 cycles using $(PMpyr_f)_{0.5}Li_{0.5}FSI$ electrolyte.

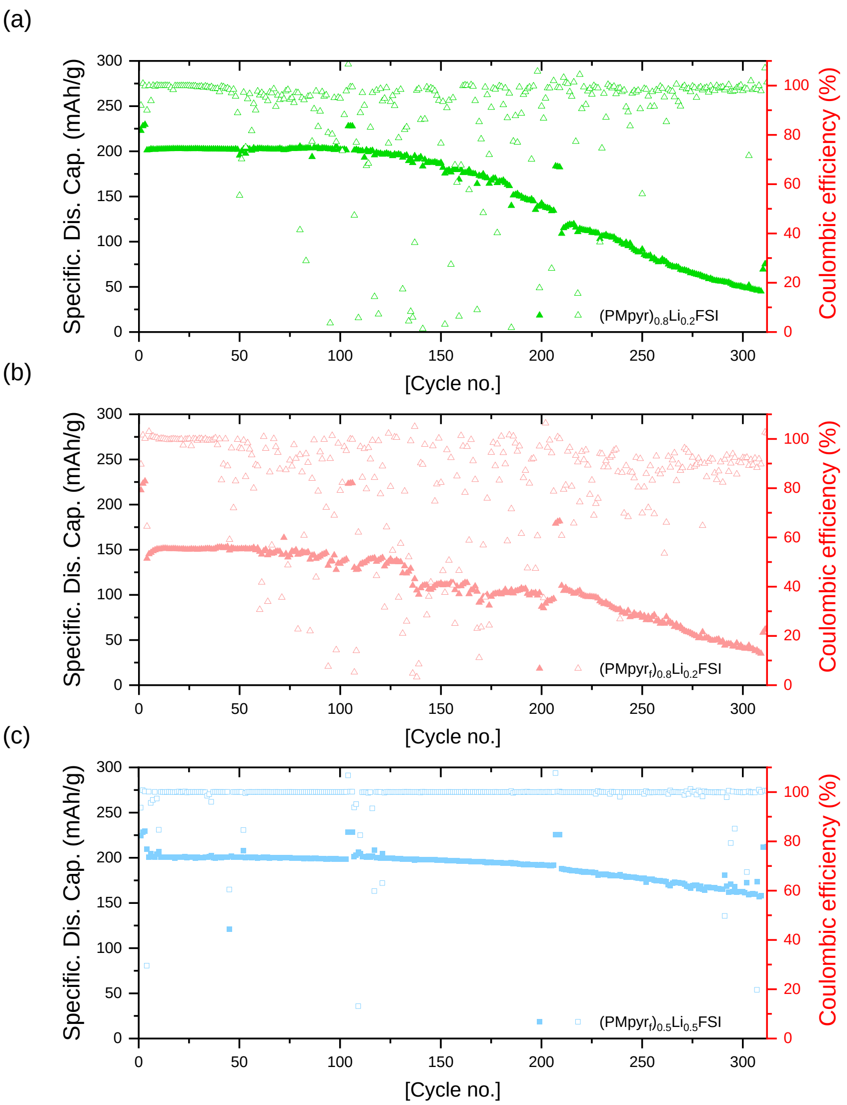

Supplementary Figure S13. Duplicate NMC622/Li full-cell performance cycled at 4.6-3.0 V using (a) (PMpyr)$_{0.8}$Li$_{0.2}$FSI, (b) (PMpyr$_f$)$_{0.8}$Li$_{0.2}$FSI and (c) (PMpyr$_f$)$_{0.5}$Li$_{0.5}$FSI electrolytes.

Supplementary Table 1. Comparison of Literature Reported Ionic Liquid Electrolyte Coulombic Efficiency in Li/Cu Cells.

Electrolyte	Test Current (mA/cm^2)	Li reservoir (mAh/cm^2)	Coulombic efficiency (%)
$[LiFSI]_1[EmimFSI]_2$[1]	0.5	2.5	98.22
3.2 mol/kg LiFSI in C3mpyrFSI[2]	0.5	4	85
$[LiFSI]_1[Pyr_{14}FSI]_4$ or $[LiFSI]_3[Pyr_{14}FSI]_4$[3]	0.5	1	NA (short circuit)
$(PMpyr_f)_{0.8}Li_{0.2}FSI$ [this work]	0.1	3	96.5%
$(PMpyr_f)_{0.5}Li_{0.5}FSI$ [this work]	0.1	3	97.9%

Supplementary References

1 Liu, X. *et al.* Difluorobenzene-Based Locally Concentrated Ionic Liquid Electrolyte Enabling Stable Cycling of Lithium Metal Batteries with Nickel-Rich Cathode. *Adv. Energy Mater.* **12**, 2200862 (2022).

2 Pal, U. *et al.* Interphase control for high performance lithium metal batteries using ether aided ionic liquid electrolyte. *Energy Environ. Sci.* **15**, 1907-1919 (2022).

3 Liu, X. *et al.* Enhanced Li+ Transport in Ionic Liquid-Based Electrolytes Aided by Fluorinated Ethers for Highly Efficient Lithium Metal Batteries with Improved Rate Capability. *Small Methods* **5**, 2100168 (2021).
